# Laser-Induced Photothermal Hydrogels Promote the Proliferation of MC3T3-E1 Preosteoblasts for Enhanced Bone Healing

**DOI:** 10.3390/jfb16020063

**Published:** 2025-02-12

**Authors:** Audrey L. Wu, Abigail F. Wu, Chieh-Ying Chen, Ruaina Lily Hope Moreno, Jia-Lin Wu, Pei-Chun Wong

**Affiliations:** 1 Graduate Institute of Biomedical Optomechatronics, College of Biomedical Engineering, Taipei Medical University, Taipei 11031, Taiwanwua25@ma.org.tw (A.F.W.);; 2 Morrison Academy Taipei, New Taipei 24449, Taiwan; 3 School of Biomedical Engineering, College of Biomedical Engineering, Taipei Medical University, Taipei 11031, Taiwan; 4 Department of Orthopedics, Taipei Medical University Hospital, Taipei 11031, Taiwan; 5 Department of Orthopedics, School of Medicine, College of Medicine, Taipei Medical University, Taipei 11031, Taiwan; 6 Orthopedics Research Center, Taipei Medical University Hospital, Taipei 11031, Taiwan; 7 Centers for Regional Anesthesia and Pain Medicine, Wan Fang Hospital, Taipei Medical University, Taipei 11696, Taiwan

**Keywords:** bone healing, alginate, gold nanoparticles, photothermal effect

## Abstract

The nonunion and delayed union of bones are common challenges in orthopedic surgery, even when bone alignment is correct and sufficient mechanical stability is provided. To address this, artificial bone grafts are often applied to fracture gaps or defect sites to promote osteogenesis and enhance bone healing. In this study, we developed an alginate-based hydrogel incorporating gold nanoparticles (AuNPs) to enhance cell proliferation and facilitate bone healing through a photothermal effect induced by near-infrared (NIR) laser irradiation. The temperature was controlled by adjusting the AuNP content. The hydrogel’s properties were characterized and cell viability was assessed. Our results indicate that while the incorporation of AuNPs slightly disrupted the hydrogel’s cross-linking network at low concentrations, cell viability remained unaffected across both low and high AuNP contents. These findings suggest that this photothermal hydrogel holds great promise for orthopedic applications to improve bone healing.

## 1. Introduction

After sustaining a bone fracture, it is commonly desired to have a speedy and healthy recovery without nonunion or incomplete healing. Bone healing starts with a hematoma that is created to act as a temporary scaffold for cell differentiation into tissue, cartilage, and eventually bone [1]. Next, through intermembranous ossification and endochondral ossification, osteoblasts and chondrocytes are differentiated, creating bone tissue and cartilage, respectively [1]. Hydrogels are networked, hydrophilic, three-dimensional polymers that have the capacity to absorb large amounts of water and proliferate cell growth [2]. Other characteristics of 3D hydrogels include their desirable biocompatibility, their ability to absorb liquid exuded from inflammation, and suitable mechanical properties [3]. Since the mid-1950s, continuous groundbreaking research has been conducted on hydrogels and their immaculate abilities. Nowadays, they are used for a wide range of applications including, but not limited to, drug delivery, wound healing, and tissue engineering. In other words, hydrogels have been widely used for tissue regeneration in bone repair [4].

The ideal temperature for DNA synthesis in BMSC is 39–41 °C [5]. With regard to the relationship between heat stimulation and osteogenesis, previous studies have shown that there is a correlation between the two, with appropriate temperatures promoting enhanced osteogenesis [6]. To achieve this ideal temperature, a safe and effective external stimulus is through light stimulation, or in our case, photothermal therapy. Recently, photothermal therapy has been used in many dimensions of medicine, including cancer treatment, melanomas, rheumatoids, etc. [7]. To raise the temperature of the scaffold and material, nanoparticles are mixed in as they absorb light, causing increased efficiency in converting light energy into heat [8]. By externally focusing the laser on the tissue inside the body, nanoparticles help the material reach the ideal temperature efficiently and strategically.

Alginate, a natural polysaccharide renowned for its excellent biocompatibility, has garnered significant attention in various fields. Alginate-based hydrogels are extensively utilized in regenerative medicine [9,10,11], drug delivery systems [12], cell carriers [13], wound healing [14], and tissue regeneration [15]. Beyond biomedical applications, these hydrogels have also found use in environmental applications [16,17], flexible electronics [18,19], and the food industry [20,21,22]. A common method for cross-linking alginate hydrogels involves the use of calcium chloride (CaCl_2_). However, CaCl_2_ induces rapid gelation, which often results in heterogeneous cross-linking within the gels [23]. In contrast, alginate hydrogels that undergo slower gelation exhibit improved structural uniformity and a higher elastic modulus, making them more desirable for applications requiring consistent mechanical properties [24].

Gold (Au), a noble metal renowned for its resistance to corrosion and oxidation, has been utilized since the Middle Ages for the treatment and diagnosis of diseases [25]. Recent advancements in nanomedicine have highlighted Au’s potential in therapeutic drug delivery and as a therapeutic agent. Colloidal Au can be covalently linked to adenoviral vectors for targeted cancer therapy and can induce hyperthermia through the application of near-infrared (NIR) laser light [26]. This is due to the unique localized surface plasmon resonance (LSPR) exhibited by colloidal Au when specific wavelengths of light interact with electrons on its surface. Modern innovations in the design of gold nanoparticles allow for localized heat generation near cancer tissues and enable controlled and targeted drug delivery. The benefits of gold nanoparticles (AuNPs) for photothermal cancer treatment include: (1) precise administration into the tumor site, minimizing non-specific distribution; (2) activation by NIR laser light, allowing deep tissue penetration; (3) versatility in creating multifaceted cancer photothermal therapy (PTT) and drug delivery systems [27]; and (4) compared to gold nanorods (AuNRs), gold nanospheres (AuNSs), and gold nanocages (AuNCs), gold nanoparticles (AuNPs) exhibit a broader excitation wavelength range [28].

This study aimed to improve the quality and rate of recovery following bone injuries by leveraging photothermal therapy and the regenerative capacity of hydrogels. The use of photothermal therapy and its combination with nanoparticles can facilitate accelerated healing by increasing local temperatures, thus promoting cellular activities essential for repair. Meanwhile, hydrogels, with their ability to support tissue and cell regeneration, create an optimal environment for bone growth. By combining these two approaches, this study sought to boost the speed and effectiveness of bone recovery. Here, we report the effects of different temperatures produced by alginate-based hydrogels with different AuNPs contents induced by laser irradiation and the cell differentiation abilities of MC3T3-E1 preosteoblasts. This study distinguished itself by incorporating gold nanoparticles (AuNPs) into alginate-based hydrogels, offering a novel approach to bone healing. The photothermal properties of AuNPs, activated by near-infrared (NIR) laser irradiation, allowed for precise localized temperature control, creating an optimal environment for preosteoblast differentiation. This dual-function strategy combined the photothermal effect to enhance cellular activity with the inherent biocompatibility and regenerative capacity of alginate hydrogels, making it a promising solution for advanced bone repair.

## 2. Materials and Methods

### 2.1. Study Design

In this study, varying concentrations of AuNPs were incorporated into alginate-CaCl_2_ hydrogel. The hydrogel’s temperature was elevated using an 808 nm laser (100 mW) through the photothermal effect to investigate its impact on MC3T3-E1 preosteoblasts (kindly provided by Prof. Chih-Hwa Chen, School of Biomedical Engineering, Taipei Medical University, Taipei, Taiwan.). We aimed to achieve a mild heat shock condition within these hydrogels to enhance cell proliferation. MC3T3-E1 preosteoblasts were cultured on alginate-based hydrogel (direct contact) and cultured with a precipitate medium (indirect contact). Additionally, we examined the characterization, degradation behavior, swelling, structure, and temperature dynamics of the hydrogel.

### 2.2. Synthesis of Alginate-CaCl_2_-AuNPs Hydrogel

The Turkevich synthesis method was used to synthesize AuNPs in this study. First, 20 mg of HAuCl_4_ was suspended in 700 µL of DI water and heated to 100 °C with an additional 195 mL of DI water. After that, 3 mL of trisodium citrate dihydrate solution (12.5 mg/mL) was added gently and stirred with a magnetic stirrer bar on top of a hot plate for an hour. AuNPs were collected by centrifugation and stored in a 4 °C refrigerator for use.

Sodium alginate solution (2 wt.%) was prepared with 2 g of sodium alginate powder in 98 g of different concentrations (0, 0.5, 1, 2, and 4 mg/mL) of AuNPs DI water suspensions. Each mixed solution was stirred for 12 h at 1000 rpm to ensure it was well mixed, and vacuum pumped to remove bubbles. We added sodium alginate-AuNPs solution into a 96-well plate and removed the bubbles again. Next, we added CaCl_2_ solution (0.2 M) into the 96-well plate and covered the sodium alginate-AuNPs solution with a specific ratio (sodium alginate-AuNPs solution/CaCl_2_ solution = 4:3). After another 12 h of gelation, we collected each alginate-CaCl_2_-AuNPs hydrogel and placed it in a 4 °C refrigerator for use.

### 2.3. Analysis of Alginate-CaCl₂-AuNPs Hydrogel via DLS, FTIR, and UV-Vis-NIR

The particle size of AuNPs was detected by dynamic light scattering (DLS-DKSH, Malvern Instruments Ltd., Malvern, UK). The characterization and composition of alginate-CaCl_2_-AuNPs hydrogel were analyzed using Fourier transform infrared spectroscopy (FTIR) (Nicolet Summit Pro, Thermo Fisher Scientific, Waltham, MA, USA). UV-Vis-NIR spectrum results of alginate-CaCl_2_-AuNPs hydrogel were used for micro-spectrometer (EE2063, OtO Photonics Inc, Taichung, Taiwan) analysis.

### 2.4. Laser-Induced Temperature Change in Alginate-CaCl_2_-AuNPs Hydrogel

The relationship between the AuNPs concentration in a hydrogel and laser irradiation induction was used to measure the temperature change to analyze and find the working temperature window for the following cell culture test. For the experimental setup, we used PDMS to raise the height of the hydrogel in the 96-well plate for cell images observation using an upright microscope. In the contract, 150 µL of PDMS with the cured solution was added into the well, then 140 µL of hydrogel was added, and finally, 100 µL of culture medium. The temperature change in the hydrogel via laser (808 nm, 100 mW) irradiation was monitored and recorded by thermal couples at 10 s, 20 s, 30 s, 40 s, 50 s, 1 min, 2 min, 3 min, 4 min, 5 min, 10 min, 30 min, and 60 min.

### 2.5. Viscoelastic Behavior of Alginate-CaCl_2_-AuNPs Hydrogel

A rheometer (MCR 302, Anton-Paar, Graz, Austria) was used to analyze the dynamic viscoelastic properties of alginate-CaCl_2_-AuNPs hydrogel with a 10 mm diameter measurement plate at 37 °C. Frequency sweep mode was used to determine the storage modulus (G’) and loss modulus (G’’) of the alginate-CaCl_2_-AuNPs hydrogel. The angular frequency ranged from 0.1 to 100 rad/s, while the shear strain was held constant at 0.5%.

### 2.6. Swelling Ratio of Alginate-AuNPs Hydrogel

For the swelling test sample preparation, 70 µL of alginate-CaCl_2_-AuNPs hydrogel was added into a 96-well plate to form cylinder samples. Initial weights were first measured by electronic balance, and then samples were immersed in simulated body fluid (Hank’s solution) in a 37 °C oven. We measured and recorded the samples’ weights at 0, 2, 4, 6, 8, 12, and 24 h to calculate the swelling ratio.

### 2.7. Degradation Behavior of Alginate-CaCl_2_-AuNPs Hydrogel

The weight change measurement was used to test the degradation behavior of alginate-CaCl₂-AuNPs hydrogel. First, 70 µL of hydrogel was added into a 96-well plate to form cylindrical samples. Samples were then freeze-dried, and the initial dry weight (W_i_) was measured using an electronic balance. After immersion in Hank’s solution (Thermo Fisher Scientific, Grand Island, NY, USA) at 37 °C, at each time point (7, 14, 21, 28, 35, 42, and 49 days) samples were removed from the solution, freeze-dried again, and the dry weight (W_d_) was measured. The remaining weight was calculated as follows:

Remaining weight (%) = W_d_/W_i_ × 100% (1)

W_d_: dry sample weight at the respective period

W_i_: initial sample dry weight

### 2.8. Evaluation of the Mechanical Properties of Alginate-CaCl₂-AuNPs Hydrogel

Compression tests were used to represent mechanical properties and were performed on a material testing machine (Pin Tai Technology, Taichuang, Taiwan) at a speed of 0.1 mm/s. The dimension of test samples was 10 mm × 10 mm × 10 mm. Each test ended when the deformation of a sample reached 2 mm (strain 20%). The testing protocol was adapted from ASTM D695 [29], with modifications to fit the soft hydrogel’s characteristics, including changes in sample size, deformation limits, and testing speed.

### 2.9. Cell Viability of MC3T3-E1 Preosteoblasts

The cell viability test was divided into two parts, indirect contact and direct contact. Both methods used a live/dead assay (Thermo Fisher Scientific, Waltham, MA, USA) to analyze cell viability. Calcein AM dye (Thermo Fisher Scientific, Eugene, OR, USA) (excitation/emission: 494 nm/517 nm) was used to stain live cells green, while SYTOX Deep Red dye (Thermo Fisher Scientific, Eugene, OR, USA) (excitation/emission: 660 nm/682 nm) was used to stain dead cells red. The indirect contact method was focused on testing the cell toxicity of the alginate-CaCl_2_-AuNPs hydrogels, and the direct contact method with laser irradiation was focused on understanding the photothermal effect on MC3T3-E1 preosteoblasts.

#### 2.9.1. Indirect Contact Method

Different groups of alginate-CaCl_2_-AuNPs hydrogels precipitate media were prepared for the indirect contact cell viability test. Each sample was immersed in αMEM medium (Gibco, Carlsbad, CA, USA) for 7 days at a concentration of 10 mg/mL. After 7 days, the precipitate medium was collected into another tube and stored at 4 °C until use. Next, 100 µL of MC3T3-E1 preosteoblasts was seeded into a 96-well culture plate with a cell density of 3000 cells/well. We pre-cultured the cells at 37 °C in a 5% CO_2_ atmosphere for cell attachment. After 24 h, we replaced the standard culture medium with different groups of precipitate media for further culturing. At days 1, 3, and 7, we stained cells with live/dead reagents and captured images using a fluorescent upright microscope (BX53, Olympus, Tokyo, Japan). Live cells were stained green and dead cells were stained red.

#### 2.9.2. Direct Contact Method with Laser Irradiation

Live/dead assays were used to test the biocompatibility of MC3T3-E1 preosteoblasts that were simulated by alginate-CaCl_2_-AuNPs hydrogels with laser irradiation. After placing 150 µL of PDMS and 70 µL of alginate-CaCl_2_-AuNPs hydrogel into a 96-well plate, we added 100 µL of MC3T3-E1 preosteoblasts in each well with a cell density of 1000 cells/well. We pre-cultured these cells for 4 h at 37 °C in a 5% CO_2_ atmosphere for cell attachment. Laser irradiation (808 nm, 100 mW) induced the hydrogels for 30 min to increase the temperature of different hydrogels at 4 h, day 2, and day 6 post-seeding time-points. At days 1, 3, and 7, we stained cells with live/dead reagents and captured images using a fluorescent upright microscope. Live cells were stained green and dead cells were stained red.

### 2.10. Statistical Analysis

Data from this study are expressed as the mean ± standard deviation. Statistical analyses were conducted by SPSS 20 (version 20.0, IBM, Armonk, NY, USA). Data were assessed using one-way variance analysis, a post hoc Tukey test, and an independent sample *t*-test. Statistical significance was established at *p* < 0.05.

## 3. Results and Discussion

### 3.1. Characterization of Alginate-CaCl_2_-AuNPs Hydrogel

**Figure 1:**
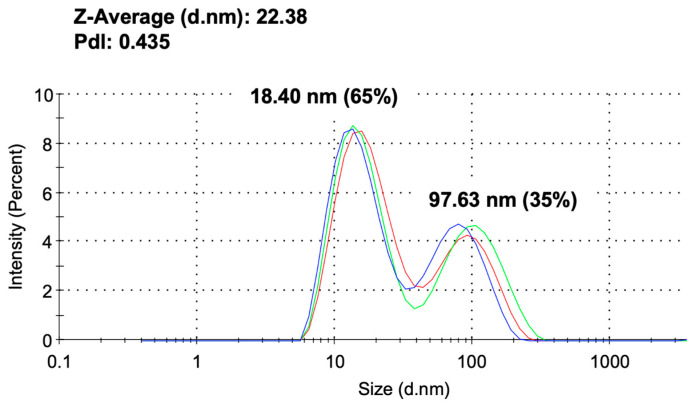
Particle size distribution of AuNPs (The three curves (blue, red, and green) represent data from three independent experimental replicates).

**Figure 2:**
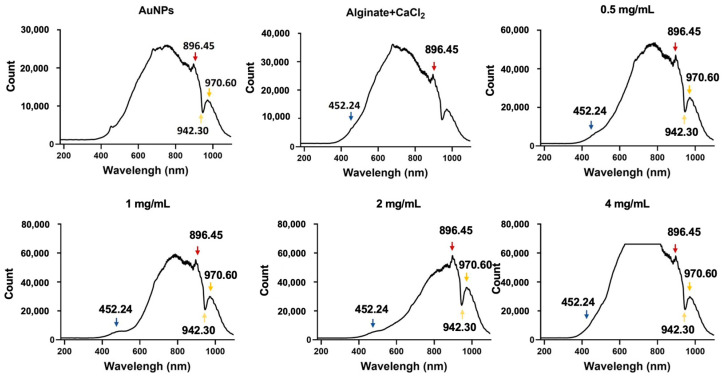
UV-visible-NIR absorption spectrum of different groups of alginate-CaCl_2_-AuNPs hydrogel (The arrows indicate specific peaks).

The particle size of AuNPs was evaluated using DLS measurements before their incorporation into alginate-CaCl₂-AuNPs hydrogel, ensuring the characterization of AuNPs alone prior to hydrogel formation. Figure 1 shows the size distribution of AuNPs without the sonication procedure. Their primary particle diameters were observed at 18.40 ± 28.1 nm and 97.63 ± 41.18 nm, with a polydispersity index (PDI) of 0.435, indicating moderate size distribution. Figure 2 presents the UV-Vis-NIR spectra of the alginate-CaCl_2_-AuNPs hydrogel. The absorption peak of AuNPs showed specific peaks at 896.4, 942.3, and 970.6 nm. With an increase in the concentration of AuNPs in the hydrogel, these specific peaks increased in intensity, which means AuNPs were well mixed into the hydrogel. A critical factor in the application of gold nanoparticles (AuNPs) in photothermal therapy (PTT) is photothermal conversion efficiency, which is influenced by the size, shape, and concentration of AuNPs in a solution [30,31]. The shape and size of these nanoparticles are particularly crucial, as they dictate the light absorption properties of the AuNPs, which subsequently convert the absorbed light into heat energy. This conversion is driven by a sequence of photophysical processes [32]. Initially, the light absorbed by AuNPs is rapidly transformed into heat, creating a hot metallic lattice. This transformation involves two key relaxation processes: electron–electron relaxation, occurring within femtoseconds, followed by electron–phonon relaxation, which takes place over picoseconds.

### 3.2. Temperature Change in Alginate-CaCl_2_-AuNPs Hydrogels During Laser Induction

**Figure 3:**
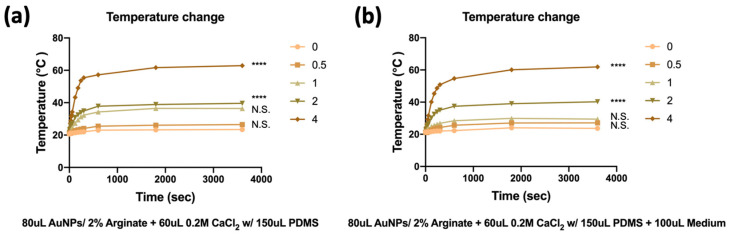
Temperature change in alginate-CaCl_2_-AuNPs hydrogel (**a**) with and (**b**) without culture medium during laser induction. (N = 5 per group; **** *p* < 0.001. N.S., not significant).

The temperature change in hydrogels after laser irradiation induction is presented in Figure 3. Figure 3a shows the temperature change in hydrogels with 150 µL of PDMS. The temperature change in hydrogels containing 0, 0.5, 1, 2, and 4 mg/mL of AuNPs demonstrated 2.8 °C, 5.5 °C, 15.4 °C, 18.0 °C, and 41.5 °C increases in temperature after 1 h of laser irradiation, respectively. These temperatures rose with increases in AuNPs content. To simulate the cell culture environment for the follow-up test, we added cell culture standard media to the culture plate using measurements shown in Figure 3b. Results were similar to results obtained without adding cell culture standard media. The temperature change in hydrogels containing 0, 0.5, 1, 2, and 4 mg/mL of AuNPs demonstrated 2.4 °C, 5.9 °C, 7.1 °C, 19.2 °C, and 40.0 °C increases in temperature after 1 h of laser irradiation, respectively. The media would not affect the hydrogel’s rising temperature by laser irradiation. The temperature rise in both groups tended to slow down after 10 min and finally showed stable temperatures of 22.4 °C, 25.9 °C, 27.1 °C, 39.2 °C, and 60.1 °C, respectively. Thus, the exposure time for laser irradiation for the follow-up in vitro test was 30 min to an hour. Heat generation by AuNPs is a result of their oscillation, known as surface plasmon resonance [33,34]. AuNPs can be engineered to accumulate in specific organs or tumors [35,36], where they generate heat to destroy cancerous tissues in a dose-dependent manner. However, we used dose control to maintain the temperature under mild heat shock (39–41 °C) for cell proliferation.

### 3.3. Viscoelastic Behavior of Alginate-CaCl_2_-AuNPs Hydrogels

**Figure 4:**
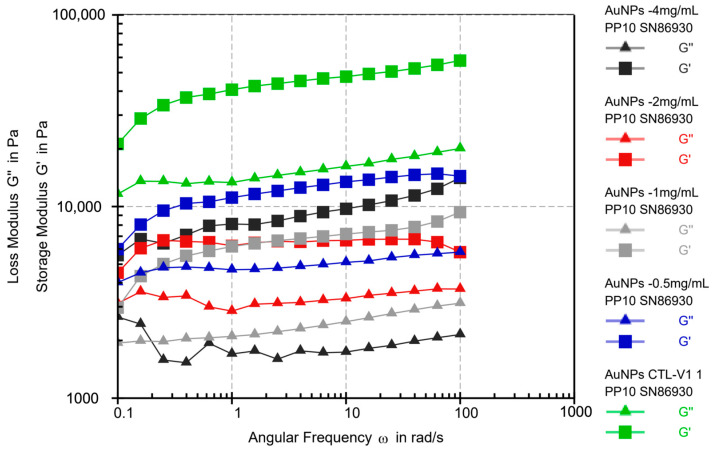
Rheological properties of different groups of alginate-CaCl_2_-AuNPs hydrogels.

The viscoelastic and viscosity behaviors of various alginate-CaCl2-AuNPs hydrogel groups were assessed using strain sweep rheological analysis post-gelation. As illustrated in Figure 4, the storage modulus (G’) was consistently higher than the loss modulus (G”), signifying that a stable and permanent gel network with an elastic (solid-like) nature prevailed over the gel’s viscous characteristics. Upon the addition of AuNPs, alginate-CaCl_2_-AuNPs hydrogels demonstrated reduced G’ and G” values, indicating the formation of fewer cross-linking networks compared to alginate-CaCl_2_ hydrogels. The strain sweep showed that alginate-CaCl_2_-AuNPs hydrogels had a smaller linear viscoelastic region (LVER), indicating they tolerated less deformation before breaking. This reduction in moduli and LVERs was likely due to AuNPs disrupting the cross-linking network. While AuNPs added functionality, they may have reduced these gels’ mechanical stability.

### 3.4. Swelling Ratio and Degradation Behavior of Alginate-CaCl_2_-AuNPs Hydrogels

**Figure 5:**
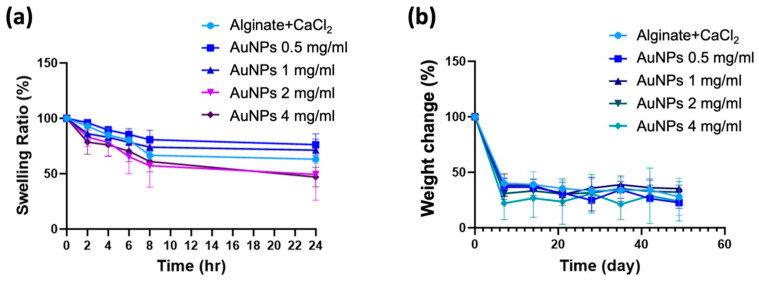
(**a**) Swelling ratio and (**b**) degradation behavior of different groups of alginate-CaCl_2_-AuNPs hydrogels immersed in simulated body solution (Hank’s solution) at 37 °C for different time periods.

The swelling ratios of different groups of alginate-CaCl_2_-AuNPs hydrogels were tested for 24 h at 37 °C, and results are shown in Figure 5a. In this swelling profile, all groups of samples presented a decreased trend. In the first 8 h, all hydrogels decreased quickly; after that, they became stable. Alginate-CaCl_2_ hydrogels with a higher AuNPs content seemed to decrease in weight more compared with the original alginate-CaCl_2_ hydrogel, which means that hydrogels containing more AuNPs had a lower swelling ability. Weight change results were used to represent the degradation behavior of alginate-CaCl_2_-AuNPs hydrogels. Figure 5b shows weight change results after normalization. At the first measurement point (7 days), alginate-CaCl_2_ hydrogel without AuNPs demonstrated the highest remaining weight, at 40.2%, and alginate-CaCl_2_ hydrogel that contained 4 mg/mL of AuNPs presented the lowest remaining weight, at 22.1%. Swelling and degradation results both indicated that the hydrogel with the higher AuNPs content showed a higher weight decrease because of weakened crosslinking. However, there were no significant differences between groups in either the swelling or degradation tests.

### 3.5. Mechanical Properties of Alginate-CaCl_2_-AuNPs Hydrogel

**Figure 6:**
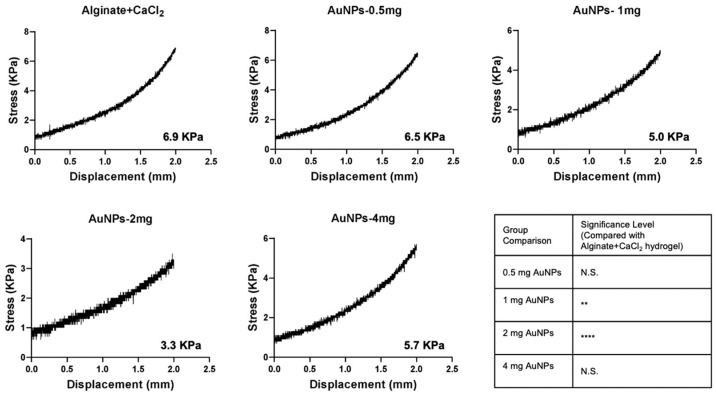
Compressive strength of different groups of alginate-CaCl_2_-AuNPs hydrogel. (N = 5 per group; ** *p* < 0.01; **** *p* < 0.001, N.S., not significant).

A compression test was used to evaluate how AuNPs affected the mechanical properties of alginate-CaCl_2_ hydrogel. Figure 6 shows that the alginate-CaCl_2_ hydrogel without AuNPs had the largest compression fracture strength of 6.9 kPa. The compression strength of alginate-CaCl_2_-AuNPs hydrogel decreased with a AuNPs concentration increase. However, the compression strength of hydrogel increased to 5.7 KPa at the AuNPs concentration of 4 mg/mL. As observed in the rheological results, AuNPs disrupted the cross-linking network, leading to a decrease in compressive fracture strength. This disruption was likely due to AuNPs interfering with the uniform distribution of cross-linking points, resulting in reduced network density and structural integrity. Consequently, the load-bearing capacity of the hydrogel may have been compromised. However, weakened bonds offer distinctive characteristics, such as reversibility, shear-thinning properties, and responsiveness to external stimuli, making them highly beneficial for biomedical applications. Physical hydrogels crosslinked by weak interactions, such as ionic bonds, demonstrate shear-thinning behavior, enabling easy injection through a syringe [37,38].

### 3.6. Cell Viability of MC3T3-E1 Preosteobasts

**Figure 7:**
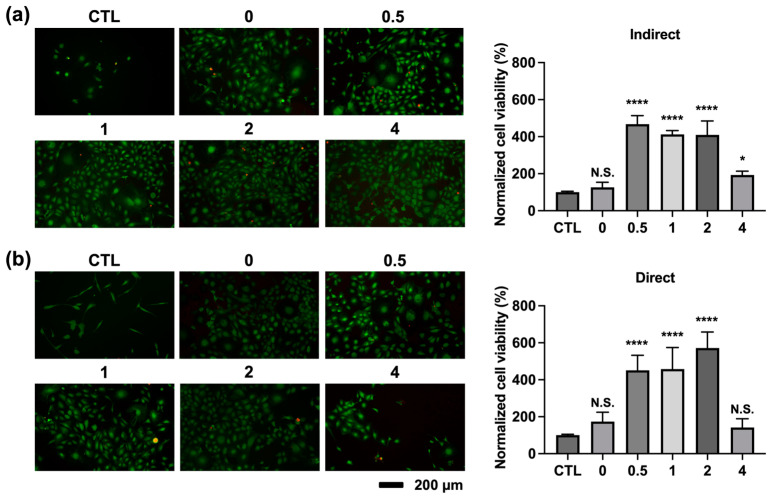
Cell viability of MC3T3-E1 preosteoblasts by live/dead assay with (**a**) indirect contact method and (**b**) direct contact method. (N = 5 per group; N.S., not significant; * *p* < 0.05; **** *p* < 0.001).

The cell viability of MC3T3-E1 preosteoblasts was evaluated using both indirect contact and direct contact methods. Figure 7 shows the results of the live/dead assay. After 7 days of incubation with precipitate media, all hydrogel groups exhibited a significantly higher cell count compared to the control group (CTL) (Figure 7a). On the other hand, Figure 7b presents the direct contact results after 7 days of incubation with different hydrogel groups and laser irradiation. Notably, the cell count in the alginate-CaCl_2_ hydrogel containing 2 mg/mL AuNPs was significantly higher than it was in the other groups, based on quantitative analysis. These results not only suggest that the incorporation of AuNPs enhanced cell viability, but also that temperature modulation by laser irradiation further promoted cell proliferation.

## 4. Conclusions

To address the challenges of bone nonunion and delayed union, we developed an alginate-based hydrogel infused with gold nanoparticles (AuNPs) exhibiting photothermal properties. The incorporation of AuNPs enhanced the chemical reactivity, modulated crosslinking density, and the mechanical properties of the hydrogel. Moreover, the photothermal properties of AuNPs enabled precise temperature regulation via laser irradiation, effectively promoting cell proliferation. Notably, an optimal AuNP concentration of 2% resulted in a remarkable increase in cell proliferation, reaching approximately 600% compared to the control group. This innovative approach represents a significant advancement in orthopedic treatment strategies and holds substantial scientific and clinical promise.

## Data Availability

The data presented in this study are available on request from the corresponding authors. The data are not publicly available since they are original data.

## Abbreviations

The following abbreviations are used in this manuscript:

AuNPs	Gold nanoparticles
CaCl_2_	Calcium chloride
NIR	Near-infrared
LSPR	Localized surface plasmon resonance
PTT	Photothermal therapy
